# Spatial-transcriptomic profiling: a new lens for understanding myelofibrosis pathophysiology

**DOI:** 10.1186/s12964-024-01877-3

**Published:** 2024-10-21

**Authors:** Edoardo Peroni, Elisabetta Calistri, Rosario Amato, Michele Gottardi, Antonio Rosato

**Affiliations:** 1 Immunology and Molecular Oncology Unit, Veneto Institute of Oncology, IOV-IRCCS, Padova, 35128 Italy; 2 Onco-Hematology, Department of Oncology, Veneto Institute of Oncology, IOV-IRCCS, Padua, 31033 Italy; 3 Medical Genetics Unit, Mater Domini University Hospital, Catanzaro, 88100 Italy; 4 Immuno-Genetics Lab, Department of Health Science, Medical School, University “Magna Graecia” of Catanzaro, Catanzaro, 88100 Italy; 5 Department of Surgery Oncology and Gastroenterology, University of Padova, Padova, 35122 Italy

**Keywords:** Myeloproliferative neoplasms, Myelofibrosis, Spatial transcriptomics, Spleen, Jak inhibitors, Personalized medicine

## Abstract

Myelofibrosis (MF) is a complex myeloproliferative neoplasm characterized by abnormal hematopoietic stem cell proliferation and subsequent bone marrow (BM) fibrosis. First documented in the late 19th century, MF has since been extensively studied to unravel its pathophysiology, clinical phenotypes, and therapeutic interventions. MF can be classified into primary and secondary forms, both driven by mutations in genes such as *JAK2*,* CALR*, and *MPL*, which activate the JAK-STAT signaling pathway. These driver mutations are frequently accompanied by additional non-driver mutations in genes like *TET2*,* SRSF2*, and *TP53*, contributing to disease complexity. The BM microenvironment, consisting of stromal cells, extracellular matrix, and cytokines such as TGF-β and TNF-α, plays a critical role in fibrosis and aberrant hematopoiesis. Clinically, MF manifests with symptoms ranging from anemia, splenomegaly, and fatigue to severe complications such as leukemic transformation. Splenomegaly, caused by extramedullary hematopoiesis, leads to abdominal discomfort and early satiety. Current therapeutic strategies include JAK inhibitors like Ruxolitinib, which target the JAK-STAT pathway, alongside supportive treatments such as blood transfusions, erythropoiesis-stimulating agents and developing combinatorial approaches. Allogeneic hematopoietic stem cell transplantation remains the only curative option, though it is limited to younger, high-risk patients. Recently approved JAK inhibitors, including Fedratinib, Pacritinib, and Momelotinib, have expanded the therapeutic landscape. Spatially Resolved Transcriptomics (SRT) has revolutionized the study of gene expression within the spatial context of tissues, providing unprecedented insights into cellular heterogeneity, spatial gene regulation, and microenvironmental interactions, including stromal-hematopoietic dynamics. SRT enables high-resolution mapping of gene expression in the BM and spleen, revealing molecular signatures, spatial heterogeneity, and pathological niches that drive disease progression. These technologies elucidate the role of the spleen in MF, highlighting its transformation into a site of abnormal hematopoietic activity, fibrotic changes, and immune cell infiltration, functioning as a “tumor surrogate.” By profiling diverse cell populations and molecular alterations within the BM and spleen, SRT facilitates a deeper understanding of MF pathophysiology, helping identify novel therapeutic targets and biomarkers. Ultimately, integrating spatial transcriptomics into MF research promises to enhance diagnostic precision and therapeutic innovation, addressing the multifaceted challenges of this disease.

## Introduction

### Myelofibrosis

Myelofibrosis (MF) stands as an intricate and challenging entity within the expansive domain of BCR::ABL1 negative myeloproliferative neoplasms (MPNs) [1]. It manifests as a disorder characterized by the aberrant proliferation of hematopoietic stem cells, precipitating a deregulated cascade in the production of blood cells [2]. Having been initially acknowledged over a century ago in 1879 with Gustav Heuck [3], MF has been the subject of exhaustive research endeavors that have substantially augmented our comprehension of its pathophysiological underpinnings, clinical phenotypes, and therapeutic modalities. This complex pathology not only poses formidable clinical challenges but also serves as an archetypal model for investigating the sophisticated interplay between genetic determinants, the bone marrow (BM) microenvironment, intricate interactions with visceral organs such as the spleen, and the orchestrating influence of the immune system.

Central to the pathology of MF is the distinctive development of fibrous tissue within the BM milieu, compromising the physiological architecture imperative for hematopoiesis. This fibrotic metamorphosis precipitates a myriad of clinical complications ranging from anemia and splenomegaly to an augmented propensity for leukemic transformation [4] as shown in the following diagram.

**Fig. 1:**
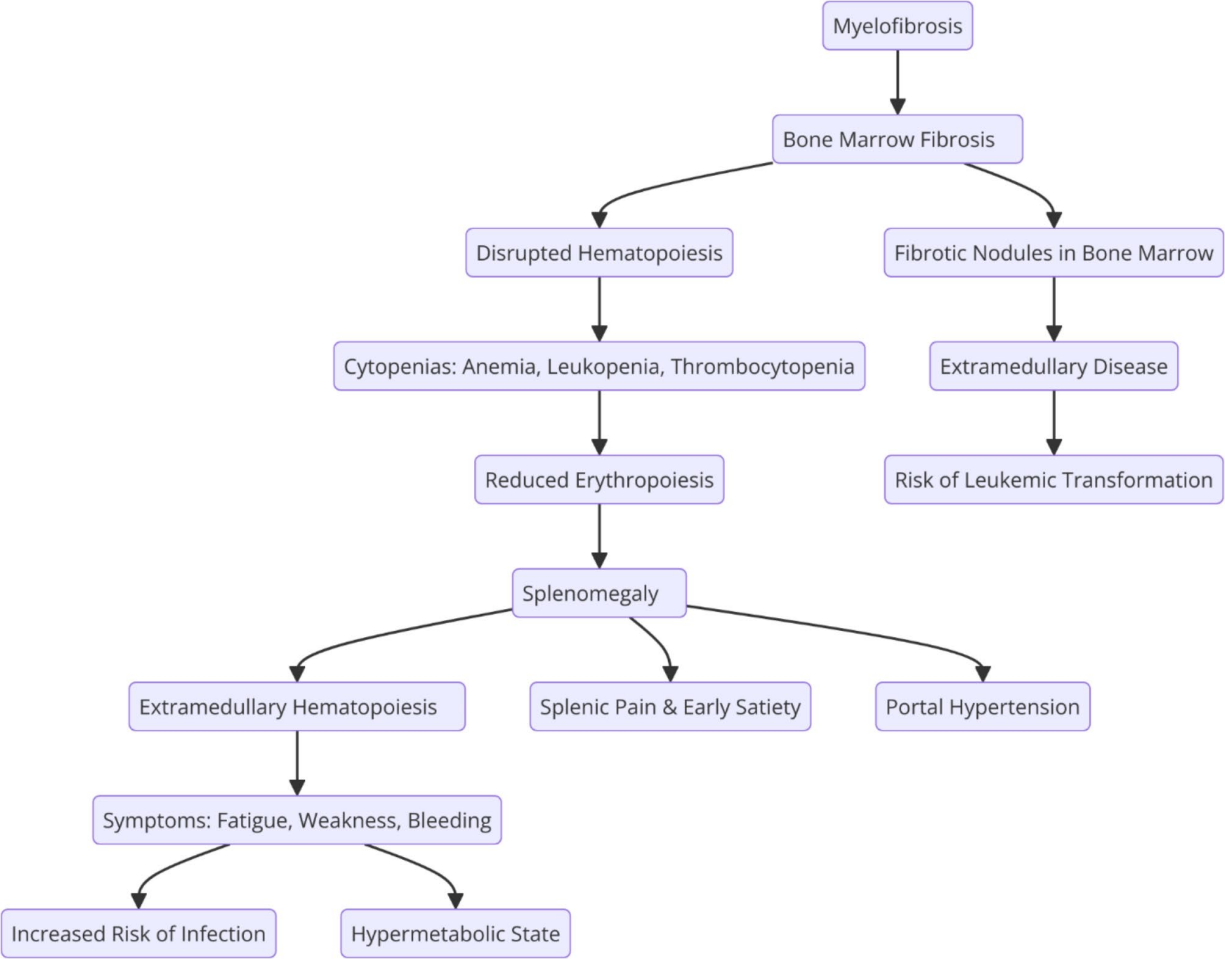
Pathophysiological relationship between MF, BM fibrosis, and splenomegaly. The diagram illustrates the progression from BM fibrosis leading to disrupted hematopoiesis, compensatory splenic involvement with extramedullary hematopoiesis, and the subsequent clinical manifestations, including cytopenias, splenic enlargement, and increased risk of leukemic transformation

This disorder is dichotomously classified into Primary Myelofibrosis (PMF), originating de novo without antecedent myeloproliferative conditions, and Secondary Myelofibrosis, evolving as a sequel from prior myeloproliferative disorders such as Polycythemia Vera or Essential Thrombocythemia [1, 5].

Predominant among the identified genetic mutations instigating MF are those affecting *JAK2*,* CALR*, and *MPL* genes [6]. These driver mutations activate signaling pathways pivotal to hematopoiesis, consequently fostering unbridled proliferation of hematopoietic stem and progenitor cells. The JAK-STAT pathway, particularly accentuated by the landmark discovery of the JAK2V617F mutation [7], represents a watershed moment in comprehending the molecular substrates of MPNs, including MF.

To note that a spectrum of additional non-driver genetic mutations imparts considerable features to the disease’s progression and prognosis, for instance genes implicated in splicing factor *ZRSR2*,* SRSF2*,* SF3B1* [8], epigenetic *DNMT3A*,* TET2*, metabolic *IDH1*,* IDH2* [9], and other pathways *TP53*,* KRAS*,* NRAS* [10, 11], . Furthermore, the dynamic interplay between hematopoietic cells and the BM microenvironment, consisting of stromal cells, extracellular matrix (ECM), and a milieu of cytokines, emerges as a pivotal factor in MF pathogenesis. Aberrant interactions contribute significantly to the fibrotic transformation. Noteworthy cytokines implicated in BM homeostasis deregulation encompass Transforming Growth Factor-beta (TGF-β), Fibroblast Growth Factor (FGF) [12], and proinflammatory cytokines like Tumor Necrosis Factor-alpha (TNF-α) [13].

Recent research has significantly expanded our understanding of how non-driver mutations influence the progression and transformation of MF, particularly PMF. Non-driver mutations, such as those in *ASXL1*, *EZH2*, *TET2*, *SRSF2*, and *IDH1/IDH2*, have been found to exacerbate disease severity, accelerate progression, and increase the risk of transformation to acute myeloid leukemia (AML).

These studies have shown that mutations in genes like *ASXL1* and *EZH2* are linked to faster disease progression, higher degrees of BM fibrosis, and shorter survival rates. These mutations tend to drive clonal evolution, contributing to genetic complexity and heterogeneity in the disease, which worsens the overall prognosis [14, 15]. *ASXL1* mutations, for instance, are associated with a more aggressive disease course, leading to poor response to therapies such as JAK inhibitors and a higher likelihood of leukemic transformation [16]. Moreover, mutations in *SRSF2* and *IDH1/IDH2* have been linked to impaired cell differentiation and increased myeloproliferation, further exacerbating the risk of transition to AML [17]. The presence of *EZH2* mutations has been found to disrupt epigenetic regulation, accelerating both fibrosis and leukemic transformation, often marking patients for worse outcomes [15].

The accumulation of non-driver mutations also directly influences treatment response. Patients harboring mutations in genes such as *ASXL1*,* SRSF2*, and *U2AF1* often show resistance to JAK inhibitors like Ruxolitinib, requiring alternative therapeutic strategies, such as combination therapies targeting epigenetic or splicing mechanisms [15]. Importantly, the number of mutations also plays a role: a higher mutational burden, particularly involving multiple high-risk mutations, is associated with increased chances of disease progression and leukemic transformation [17]. This makes comprehensive genetic testing crucial for risk stratification, as identifying these mutations can guide personalized treatment approaches aimed at mitigating disease progression and improving patient outcomes. A comprehensive study conducted across 60 institutions in Spain analyzed the impact of these mutations in 312 patients with PMF using targeted next-generation sequencing (NGS). This study revealed that 72% of patients had non-driver mutations, with 47% harboring two or more mutations. The most frequent mutations detected were in *ASXL1* (34%), *TET2* (22%), *SRSF2* (17%), *U2AF1 Q157* (9%), *CBL* (8%), *EZH2* (7%), *TP53* (6%), *DNMT3A* (6%), and *SETBP1* (5%). The presence of these mutations was strongly associated with worse overall survival (OS). Specifically, patients without mutations had a median OS of 12.4 years, whereas those with one mutation had a median OS of 8.7 years, and those with two or more mutations had a significantly reduced median OS of 4.4 years (*P* < .001) [18].

These findings underscore the adverse prognostic impact of non-driver mutations, which contribute to poorer clinical outcomes and higher mortality rates. Notably, *ASXL1* mutations emerged as the most prevalent high-risk mutations, with a variant allele frequency > 20% identified as a critical adverse prognostic factor, particularly influencing decisions regarding allogeneic hematopoietic stem cell transplantation (allo-HSCT). The study also highlighted the independent prognostic significance of *TP53*,* CBL*, and *SETBP1* mutations, advocating for their inclusion in the Molecular International Prognostic Scoring System (MIPSS70 and MIPSS70 plus) models for PMF. This aligns with previous findings suggesting that mutations in these genes should be considered in prognostic models to better stratify patients and tailor treatment strategies [19, 20]. Overall, the integration of non-driver mutations into clinical practice is crucial for improving risk stratification and guiding personalized treatment approaches in MF. These mutations not only exacerbate the disease course but also complicate treatment, often leading to resistance to therapies like JAK inhibitors, and accelerating progression to more severe stages, including AML [14–16].

MF, embodying a formidable and multifaceted clinical entity, epitomizes the intricate symbiosis between genetic mutations, deregulated signaling pathways and aberrant interactions between hematopoietic and stromal cells within the BM microenvironment and extramedullary niches [21]. The evolving comprehension of its molecular foundations has smoothed the path for targeted therapeutic modalities, instilling optimism for enhanced disease management [22]. Nevertheless, the intricate nature of MF continues to propel ongoing research endeavors, aiming to unravel deeper insights into its pathogenesis and foster innovative therapeutic strategies to effectively address the diverse clinical challenges posed by this malady.

The clinical manifestations of MF exhibit a spectrum of diversity, spanning from mild constitutional symptoms to severe, life-threatening complications [23]. Commonly encountered symptoms include anemia, myeloblastosis, fatigue, and weight loss, reflective of compromised normal blood cell production (Fig. 1) [24]. Organomegaly, including splenomegaly and hepatomegaly, indicative of the pervasive impact on visceral organs, is a hallmark feature that results from extramedullary hematopoiesis (EMH) and contributes to abdominal discomfort and early satiety [25]. Simultaneously, the progressive fibrosis within the BM and deviations in blood cell counts manifest as anemia, thrombocytopenia, and leukopenia, further exacerbated by cytopenia-associated bone pain and fatigue necessitating interventions such as blood transfusions or other supportive measures [26, 27].

As we pore over the intricate molecular landscape, a comprehensive understanding of these classifications, clinical features and genetic aberrations becomes pivotal for advancing diagnostic precision and therapeutic interventions in the realm of MF. The ongoing quest for unraveling the complex facets of MF underscores the imperative to refine our knowledge base, ultimately enhancing diagnostic accuracy and facilitating targeted therapeutic innovations for improved patient outcomes.

### Bone marrow niche and microenvironment

The BM niche constitutes a specialized microenvironment crucial for the maintenance and regulation of hematopoietic stem cells (HSCs). Comprising stromal cells, endothelial cells, osteoblasts, and ECM, the niche orchestrates the delicate balance between HSCs self-renewal and differentiation [28]. In MF, this equilibrium is disrupted, precipitating aberrant cellular behaviors within the BM microenvironment. Abnormal signaling from mutated hematopoietic cells prompts the proliferation of fibroblasts, culminating in heightened collagen deposition, characteristic of fibrosis development. Furthermore, somatic mutations in *JAK2*, *CALR*, and *MPL* disturb signaling pathways within the niche, further perturbing the intricate dance between HSCs fate decisions [29]. Particularly, the persistent JAK-STAT signaling promote the uncontrolled expansion of myeloid progenitors.

As it is known, the hallmark of MF is BM fibrosis, characterized by the deposition of fibrous tissue that disturbs normal hematopoietic architecture. Activated stromal cells, including fibroblasts and myofibroblasts, contribute to excessive production and deposition of ECM proteins, notably collagen. The upregulation of TGF-β further stimulates fibroblast activation [30]. Important to mention alterations in the interactions between hematopoietic cells and the ECM that contribute to the fibrotic process. Actually, changes in the ECM’s composition and organization affect normal homing, retention, and differentiation of HSPCs [31].

Furthermore, the inflammatory microenvironment emerges as a distinguishing feature of MF, marked by the deregulated production of cytokines and chemokines affecting both hematopoietic and stromal cells [29, 32]. Immune deregulation within the microenvironment, with abnormal activation of immune cells, may contribute to disease pathogenesis and progression. In statement inflammatory cytokines, such as TNF-α, IL-6, and IL-1, are elevated in MF, disrupting the BM microenvironment [33].

These cytokines promote the vicious circle where stimulating fibrotic factor production, enhance mutant cell survival, and contribute to a pro-inflammatory milieu that sustains the disease.

Epigenetic changes, particularly altered DNA methylation patterns, further compound the complexity, influencing gene expression dynamics in both hematopoietic and stromal cells [34].

MF is linked to angiogenic changes in the BM vasculature, causing disruptions in normal vascular architecture and contributing to hypoxia. Key regulators of angiogenesis, including angiopoietin-1 and angiopoietin-2, are deregulated [13].

For these reasons MF involves a complex interplay of genetic mutations, deregulated signaling pathways, and changes in the BM microenvironment. Unraveling these intricacies is essential for developing effective interventions that address the root causes and improve outcomes for individuals affected by MF.

### Role of the spleen in myelofibrosis

Splenomegaly is a prominent feature in the landscape of MF, driven by numerous alterations in the hematopoietic system [35]. Excessive EMH, typically confined to the BM, extends to the spleen due to the perturbed BM microenvironment [36]. This expansion is further accentuated by the spleen’s pivotal role as a reservoir for blood cells, especially red blood cells, compensating for compromised production in the altered BM. Pathological changes within the spleen mirror those observed in the BM, featuring the development of fibrosis and consequential alterations in the microenvironment, potentially impairing normal splenic functions [5].

The spleen plays a crucial role in the pathophysiology of MF, acting as a reservoir for malignant HSCs that retain a full differentiation program. This suggests that the spleen is not only a site of EMH but also significantly contributes to the maintenance and progression of the disease. The interaction between these malignant HSCs and the splenic microenvironment, particularly mesenchymal stromal cells (MSCs), is key to regulating this aberrant hematopoiesis. MSCs in the spleen provide a supportive niche that enables the survival and proliferation of these malignant cells, thereby promoting disease progression [37, 38].

**Fig. 2:**
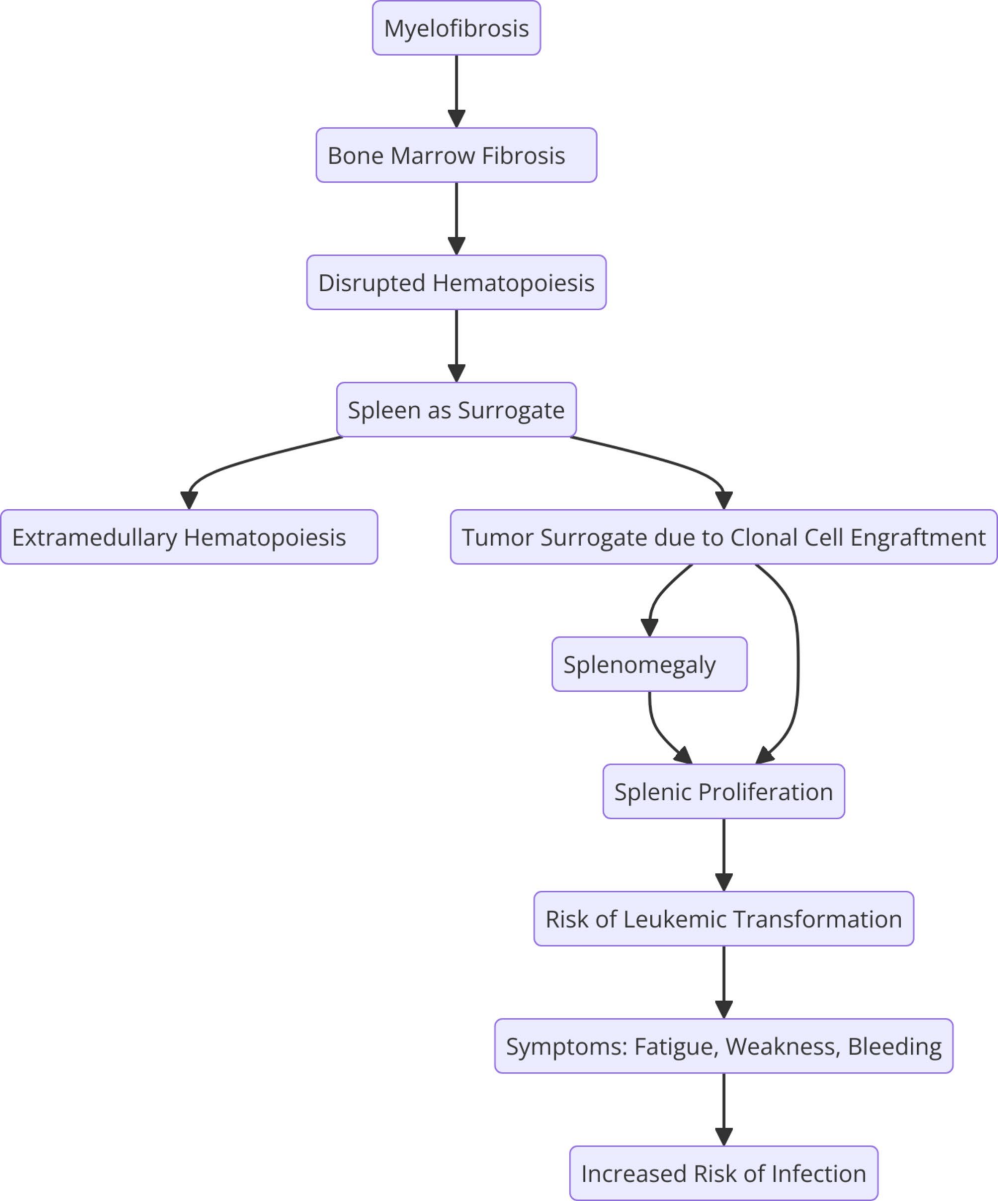
Representation of the spleen’s role as a tumor surrogate in MF due to the engraftment of clonal cells. This diagram emphasizes the spleen’s compensatory function in hematopoiesis, leading to splenomegaly, EMH, and potential leukemic transformation, underscoring the spleen’s involvement in disease progression

Our preliminary data, presented at the EHA Congress 2024, further elucidate the complex molecular landscape of the spleen in MF patients. Utilizing spatial transcriptomic analyses on spleen specimens from MF patients, we observed a modulated gradient of gene expression across distinct spleen regions. Specifically, there is a decremental gradient in gene expression related to inflammation and ECM remodeling from intravascular to perivascular regions, culminating in the red pulp. Conversely, an incremental expression gradient is noted in genes associated with iron metabolism, hemoglobin synthesis, oxygen transport, and erythropoiesis [39]. These findings suggest a dynamic orchestration of inflammatory responses, ECM alterations, and EMH within the spleen, characterizing MF and we can mint the concept of the spleen as a “tumor surrogate”. In fact in MF the spleen encapsulates its transformation into a site of massive infiltration by abnormal hematopoietic cells, especially in advanced disease stages (Fig. 2). While metaphorically labelled a “tumor surrogate”, the spleen in MF does not adhere to the characteristics of a true neoplastic mass. Instead, this term underscores the invasive and disruptive nature of the spleen’s altered state, akin to a pathological mass, albeit without the hallmark features of a malignancy [37–39].

The findings from our preliminary study underscore the need for further investigation into targeted therapies that address the unique spatial characteristics of the splenic microenvironment in MF.

Moreover spleen volume, often used as a surrogate marker for disease response in MF, reflects not only the disease burden but also serves as an indirect measure of tumor activity and the splenic microenvironment’s role in sustaining the malignant clone. Changes in spleen size could directly relate to shifts in the underlying disease biology, making it a crucial parameter in both prognosis and treatment decisions [40, 41]. These integrated insights highlight the spleen’s dual role in sustaining the disease and serving as a measurable indicator of therapeutic efficacy in MF, making it a focal point in both understanding and managing the disease.

Clinically, the consequences of splenomegaly in MF extend beyond mere organ enlargement. Symptoms such as abdominal discomfort, early satiety, and pain often arise, reflecting the physical consequences of an enlarged spleen. Furthermore, the heightened sequestration activity of the spleen exacerbates cytopenia, intensifying anemia and other blood cell deficiencies [42]. In therapeutic considerations, splenectomy emerges as a potential intervention to alleviate symptoms and ameliorate blood counts in select cases [43]. However, this approach is not without risks and is generally reserved for specific situations where the benefits outweigh potential complications. The labyrinthine role of the spleen in MF necessitates a deeper understanding of its pathophysiological transformations for the formulation of targeted and effective therapeutic strategies.

### Therapeutic approaches

Therapeutic interventions for MF are designed to address the fundamental pathogenic mechanisms, alleviate symptomatic burden, and enhance the overall well-being of afflicted individuals. Among the pivotal strategies employed, JAK inhibitors, with Ruxolitinib at the forefront, assume a central role by targeting the deregulated JAK-STAT signaling pathway [44]. By mitigating constitutional symptoms, reducing splenomegaly, and enhancing quality of life, these inhibitors represent a significant advancement in disease management, particularly for those classified as intermediate- and high-risk. Beyond JAK inhibition, supportive care measures encompass interventions such as blood transfusions, erythropoiesis-stimulating agents, androgens, immunomodulatory agents (Thalidomide, Lenalidomide), and Prednisone which prove essential in managing anemia [45], whereas the use of Hydroxyurea (Carbamide) to manage leukocytosis and thrombocytosis, contribute to holistic disease management [46]. Allo-HSCT stands as the singular curative option, albeit constrained by factors like age and comorbidities, primarily reserved for younger patients with high-risk profiles [47]. Notably, newer JAK inhibitors, such as Fedratinib and Pacritinib, provide alternative therapeutic avenues, showcasing efficacy in specific patient subsets [48, 49]. A recently approved JAK inhibitor is Momelotinib which is an ATP-competitive small molecule inhibitor, that exerts its action by selectively targeting JAK1, JAK2 or JAK3, and TYK2 protein kinases, while also modulating activin A receptor type 1 (ACVR1), also known as activin receptor-like kinase 2 (ALK2), which represent pivotal clinical targets [50]. In Europe, the authorization of Momelotinib was granted on January 29, 2024, based on data derived from the MOMENTUM (NCT04173494) pivotal phase III trial [51], alongside a cohort of adult patients displaying moderate to severe anemia (hemoglobin < 10 g/dL) extracted from the SIMPLIFY-1 (NCT01969838) phase III trial [52]. Notably, Momelotinib secured approval from the FDA on September 15, 2023 [53], predicated on data emanating from same clinical investigations. This regulatory green light was for the treatment of anaemic patients afflicted with high/intermediate risk MF, encompassing both primary and secondary MF variants [54].

The exploration of combination therapies involving Ruxolitinib alongside histone deacetylase inhibitors or immunomodulatory drugs underscores ongoing endeavors to optimize treatment outcomes [55]. Combination therapies for MF are being actively investigated to enhance treatment efficacy by targeting multiple pathways involved in the disease. JAK inhibitors, such as Ruxolitinib, remain a key component of therapy, often used in combination with other novel agents to improve patient outcomes. For example, combining Ruxolitinib with Selinexor, an XPO1 inhibitor, has shown promise in reducing spleen size and alleviating symptoms by simultaneously targeting the JAK-STAT and nuclear export pathways [56]. Similarly, the combination of Ruxolitinib with Pelabresib (CPI-0610), a BET inhibitor, targets epigenetic regulation and has demonstrated improved responses in patients who are refractory to JAK inhibitors alone [57]. Other promising strategies include combining Ruxolitinib with Navitoclax, a BCL2 inhibitor, to enhance apoptosis and reduce spleen size, particularly in patients with resistance to Ruxolitinib monotherapy [58]. In addition, antifibrotic agents like PRM-151 are being tested with Ruxolitinib to target both inflammatory and fibrotic components of the disease, showing potential to reduce BM fibrosis and improve blood counts [59]. Furthermore, immune modulators such as Pomalidomide combined with Ruxolitinib have demonstrated efficacy in managing anemia and reducing transfusion needs in patients with severe cytopenias [60]. Lastly, hypomethylating agents like Azacitidine or Decitabine, when used with JAK inhibitors, have shown promise in improving BM function and potentially modifying disease progression [59]. These combination therapies represent a comprehensive approach to managing MF, targeting various aspects of the disease to provide better outcomes for patients.

As well, ongoing research, including clinical trials, probes into novel agents and targeted therapies tailored to specific mutations or anti-fibrotic mechanisms [61]. Symptomatic management, encompassing pain control and anticoagulation due to heightened thrombotic risk, complements the therapeutic paradigm. Rigorous disease monitoring, entailing periodic assessments of blood counts, molecular profiles, and imaging studies, remains integral for gauging disease progression and treatment response. In the evolving landscape of MF therapeutics, a variegated and personalized approach is imperative, considering diverse patient characteristics and the burgeoning insights into the intricacies of the disease.

The intricate interplay between the BM niche and MF pathophysiology underscores the mottled nature of therapeutic strategies, necessitating a comprehensive understanding of the molecular intricacies within the microenvironment for effective clinical interventions.

## Spatial transcriptomic

Spatially Resolved Transcriptomics (SRT) is a cutting-edge approach in molecular biology that has revolutionized our ability to analyze gene expression patterns within the context of spatial organization in tissues. This method has emerged as a powerful tool for unraveling the intricacies of cellular heterogeneity, microenvironmental interactions, and the spatial distribution of genetic information within complex biological systems [62].

**Fig. 3:**
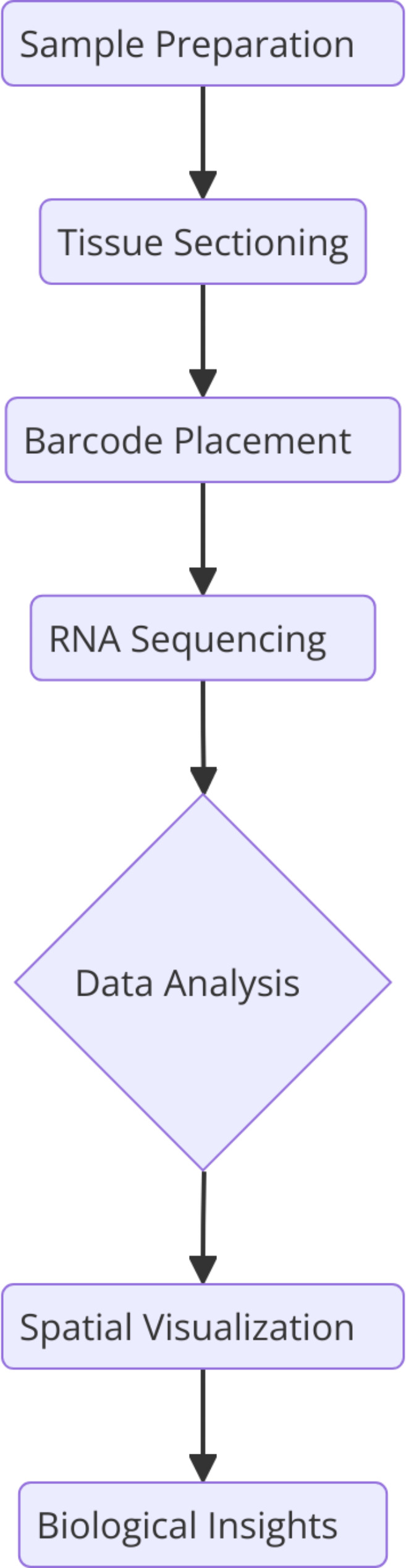
Overview of the spatial transcriptomics workflow, detailing the key steps from sample collection to data analysis and biological interpretation. The diagram highlights the integration of various advanced technologies such as CosMX, GeoMX, Xenium, HybISS, seq-FISH, and Slide-seq/Slide-seqV2, etc. in the spatial mapping of gene expression within tissue samples

Traditional transcriptomics methods, such as bulk RNA sequencing, provide valuable insights into overall gene expression within a tissue but lack the spatial resolution necessary to discern the specific location of transcriptional activities.

During the last decade Single-cell RNA sequencing (scRNA-seq) has emerged as a powerful technology that enables the profiling of gene expression at the individual cell level, providing unprecedented insights into cellular heterogeneity within tissues [63]. This technique allows researchers to analyze the transcriptomes of thousands to millions of individual cells simultaneously, identifying distinct cell types, states, and lineages. By capturing the diversity of gene expression across different cells, scRNA-seq has revolutionized our understanding of complex biological processes, such as development, immune responses, and disease mechanisms, particularly in heterogeneous tissues like tumors. The ability to dissect cellular heterogeneity at this resolution has made scRNA-seq an invaluable tool in fields ranging from cancer research to neuroscience [64].

While scRNA-seq provides deep insights into the gene expression profiles of individual cells, it lacks the ability to retain spatial information about the cells within their native tissue context. This limitation is where spatial transcriptomic technologies play a crucial role. Spatial transcriptomics preserves the spatial organization of gene expression, enabling researchers to study how cells interact within their native environment. Unlike scRNA-seq, which dissociates cells from their tissue context, spatial transcriptomics retains the architectural features of tissues, allowing for the mapping of gene expression patterns in situ.

For instance, a study on cancer tissues demonstrated the complementary nature of these technologies: scRNA-seq identified diverse cell populations within the tumor microenvironment, while spatial transcriptomics provided critical insights into how these populations were spatially organized and interacted within the tissue. This combined approach revealed how tumor cells communicate with their microenvironment, contributing to tumor progression [65].

The main advantage of scRNA-seq lies in its ability to capture the full transcriptome of individual cells, offering high-resolution data on cellular heterogeneity. However, this comes at the cost of losing spatial information, which is crucial for understanding how cell positioning and interactions influence gene expression and function. On the other hand, spatial transcriptomics offers lower resolution at the single-cell level but provides critical spatial information, making it ideal for studying tissue architecture and microenvironmental influences. ScRNA-seq excels in uncovering cellular diversity and identifying novel cell types, while spatial transcriptomics is essential for understanding the spatial organization and interaction of cells within tissues. These technologies are complementary, and their combined use can offer a more comprehensive understanding of complex biological systems [65, 66].

SRT seeks to overcome other transcriptomics technologies limitations by preserving the spatial context of gene expression, allowing researchers to explore how specific genes are regulated within distinct regions of tissues or organs [67].

SRT techniques employ a spatially barcoded microarray or bead-based platform to capture and quantify RNA molecules directly from tissue sections [68]. Each spatially resolved element on the platform corresponds to a specific location within the tissue, allowing for the simultaneous profiling of numerous genes in a spatially defined manner. This high-throughput method enables the generation of detailed spatial transcriptomic maps, enlightening the molecular landscape of tissues with unprecedented resolution (Fig. 3) [69].

The significance of spatial transcriptomics becomes particularly apparent when studying complex biological processes, such as development, disease progression, or response to therapeutic interventions. It enables researchers to investigate how gene expression patterns vary across different cell types, compartments, or regions within a tissue, providing a holistic understanding of the spatial heterogeneity inherent in biological organization [70].

In the sphere of disease research, including cancer biology and neurobiology, spatial transcriptomics has unveiled novel insights into the molecular underpinnings of pathological conditions. It allows for the identification of specific cell types contributing to disease progression, the exploration of immune responses within the tumor microenvironment, and the mapping of intricate signaling networks in neural tissues [71].

Moreover, the integration of spatial transcriptomic data with other omics technologies, such as genomics, proteomics, and metabolomics, enhances our ability to comprehend the complexity of biological systems comprehensively. This integrated approach provides a multidimensional view of spatially resolved molecular information, paving the way for a more refined understanding of biological phenomena [72, 73].

As spatial transcriptomics continues to evolve, it holds tremendous promise for advancing our knowledge in fields ranging from basic biology to clinical applications. It facilitates the discovery of novel biomarkers, the identification of potential therapeutic targets, and the development of personalized medicine approaches that account for the spatial context of gene expression. In essence, spatial transcriptomics represents a transformative paradigm in molecular biology, enabling researchers to unravel the spatial intricacies of gene regulation and offering unprecedented insights into the functional architecture of complex tissues.

This discussion inspects several SRT technologies, each contributing unique strengths and modulations to our comprehension of the disease (Table 1).

The NanoString **GeoMx Digital Spatial Profiler (DSP)** Protein and RNA assay represents a pinnacle in spatial barcoding technology, where individual mRNA or protein molecules are uniquely barcoded, allowing their identification during downstream analysis. The system utilizes high-resolution imaging, enabling a detailed analysis of cellular and molecular landscapes [74]. Its multiplexing capabilities, allowing the simultaneous profiling of hundreds of genes and proteins, provide a comprehensive spatial readout.

NanoString GeoMx RNA Assays are designed specifically for high-throughput RNA profiling. This SRT technology allows researchers spreading from the whole transcriptome atlas to customize panels for targeted gene expression analysis (cancer transcriptome atlas or immune pathways panel), accommodating various sample types, including formalin-fixed paraffin-embedded (FFPE) tissues cryopreserved and fresh biopsies and BM trephine [75].

**Hybridization-based In Situ Sequencing** (HybISS) represents a transformative approach in spatial transcriptomics by combining the precision of in situ hybridization with the power of sequencing [76]. Tissue sections undergo iterative rounds of hybridization with fluorophore-labeled probes, followed by imaging [77]. The critical innovation lies in the direct sequencing of the associated RNA sequences in situ. This unique feature provides spatially resolved information about gene expression patterns directly within intact tissues. Imagine HybISS as a molecular storyteller, capturing the essence of gene expression narratives within the spatial context of tissues. Fluorophore-labeled probes play the roles of characters in this narrative, each revealing its unique part in the story. The HybISS acts as the unfolding plot, providing a direct and intimate insight into the spatial nuances of gene expression. While HybISS preserves the spatial elegance of biological landscapes, its limitations in throughput remind us that every narrative requires careful consideration and selective storytelling to reveal the intricacies within the biological manuscript [62].

**Slide-seq/Slide-seqV2**, another SRT technology, adopts another spatial barcoding approach for high-throughput transcriptomic analysis of tissue Sect. [78]. While offering high-throughput transcriptomic profiles, it may face resolution limitations compared to other methods. Its versatility allows application across various tissues and sample types, but performance may vary depending on the sample’s size and characteristics [79].

**Sequential Fluorescent In Situ Hybridization** (SEQ-FISH) is an advanced spatial transcriptomics technique designed to unravel the spatial distribution of RNA transcripts within complex biological tissues. The method employs combinatorial fluorescence in situ hybridization, involving iterative rounds of hybridization with sets of fluorophore-labeled probes, each designed to target specific RNA sequences [80]. By sequentially imaging the resulting fluorescence signals from each round, SEQ-FISH enables the visualization of individual RNA molecules with high multiplexing capacity. SEQ-FISH is similar to an intricate molecular dance within tissues, where fluorescently labeled probes perform a meticulously orchestrated routine. These probes, similar to molecular choreographers, specifically target individual RNA sequences. The successive rounds of hybridization unveil a mesmerizing spatial tapestry of RNA distribution, analogous to unraveling the layers of a molecular canvas. While the method offers an unparalleled view into the intricate ballet of gene expression at subcellular resolution, its complexity and labor-intensive nature require researchers to navigate carefully through the nuances of manual handling and signal interpretation [81].

The 10x Genomics **Visium Spatial Gene Expression** integrates spatial barcoding with RNA sequencing, capturing both gene expression and morphological information [82]. This platform designed to map gene expression across entire tissue sections while preserving spatial context. It involves placing a tissue section on a slide that contains spatially barcoded capture spots.

These spots capture mRNA from the tissue, allowing for the spatial localization of gene expression when sequenced. Visium provides high-resolution insights into the spatial organization of gene expression, making it a valuable tool for understanding complex tissue architecture and cellular interactions in various research areas, including oncology, neuroscience, and developmental biology. This comprehensive analysis provides a balanced approach, offering insights into both transcriptomics and spatial context. However, the integrated nature of the data demands sophisticated computational analyses, and operational costs can be relatively high [83].

Additionally, **Xenium** is a spatial transcriptomics platform developed by 10x Genomics. It is designed to provide high-resolution, spatially resolved gene expression data across entire tissue sections. This platform combines the power of spatial resolution with high-throughput sequencing to map thousands of RNA molecules in situ, providing a detailed view of tissue architecture and cellular interactions. It leverages advanced imaging and sequencing technologies to capture spatially resolved transcriptomes at single-cell resolution. Tissue samples are hybridized with a set of probes that bind to target RNA molecules. These probes are then visualized through fluorescence imaging, allowing researchers to identify the precise location of each transcript. After imaging, the RNA is sequenced to provide a comprehensive gene expression profile for each spatial location within the tissue. Its ability to provide high-resolution spatial data makes Xenium an invaluable tool for advancing precision medicine and deepening our understanding of complex biological systems [84].

**CosMX Spatial Molecular Imager (SMI)** is NanoString Technologies platform for high-resolution spatial transcriptomics, enabling the visualization and quantification of gene expression at the subcellular level across entire tissue samples. This technology uses advanced multiplexed fluorescence imaging combined with proprietary in situ hybridization chemistry to detect thousands of RNA and protein targets within their native spatial context. CosMX SMI employs a sophisticated hybridization strategy, where tissue sections, either FFPE or fresh frozen, are subjected to in situ hybridization with a pool of five gene-specific probes. These probes are designed with a target-binding domain and a shared readout domain, enhancing detection sensitivity, particularly in FFPE tissues where RNA may be fragmented. The platform supports 16 iterative rounds of fluorescent imaging, during which secondary probes bind to specific subdomains of the primary probes. This process, involving a UV-cleavable linker, allows for the sequential capture and removal of fluorescent signals, enabling the simultaneous imaging and quantification of over 1000 RNA and more then 60 protein targets at subcellular resolution. The platform’s sophisticated image processing algorithms then quantify these fluorescence signals, revealing high-resolution spatial maps of gene expression relative to the tissue architecture. This spatial data is further integrated with bioinformatics analyses to uncover gene expression networks, signaling pathways, and cellular interactions. Despite some technical challenges common to imaging-based spatial technologies, such as optical crowding and long imaging times, CosMX SMI mitigates these issues through its innovative design, allowing for the profiling of more genes than other platforms [85, 86].

## Spatial proteomics

Spatial proteomics is an advanced field that combines protein analysis with spatial information, enabling researchers to map the distribution and interaction of proteins within their native tissue environments at high resolution. By integrating proteomics with spatial context, this approach provides crucial insights into how proteins influence cellular function, tissue architecture, and disease progression. Spatial proteomics technologies are particularly valuable for studying complex biological systems like the tumor microenvironment, where the spatial organization of proteins plays a critical role in understanding disease mechanisms and developing targeted therapies.

**Deep Visual Proteomics** (DVP) is a state-of-the-art technology that combines high-resolution imaging with mass spectrometry-based proteomics to analyze thousands of proteins simultaneously within tissue samples. This approach enables the precise identification and localization of proteins at the single-cell level, making it possible to explore cellular heterogeneity and the microenvironment in complex tissues like tumors. DVP captures detailed spatial proteomics data by integrating multiplexed imaging with high-throughput proteomic analysis, allowing researchers to visualize specific proteins within their native context while also quantifying them across large scales. The technology is particularly valuable for understanding how different cell types interact within their microenvironments and how these interactions contribute to disease processes. DVP’s ability to maintain spatial resolution while providing comprehensive proteomic profiles makes it an indispensable tool in modern biomedical research [87].

**Spatially Resolved Top-Down Proteomics (TDP)** is another cutting-edge technology that offers detailed proteoform identification and quantification directly from tissue sections. Unlike traditional bottom-up proteomics, TDP analyzes intact proteins, allowing for the detection of post-translational modifications and protein isoforms, which provides a deeper understanding of protein function and diversity. TDP operates at a subcellular level, enabling researchers to study the spatial distribution of different proteoforms within tissues with high precision. This technology is especially useful for investigating how various proteoforms are localized within specific cellular compartments and how these localizations influence cellular function and disease progression [88].

**Multiplexed Ion Beam Imaging** (MIBI) is a spatial proteomics technology that utilizes secondary ion mass spectrometry to achieve high-dimensional protein imaging with single-cell resolution. In MIBI, tissue sections are stained with antibodies conjugated to isotopically pure metal tags. These metal-tagged antibodies bind to their respective protein targets within the tissue. An ion beam is then used to ablate the tissue surface, releasing the metal ions, which are detected and quantified by a mass spectrometer. This method allows for the simultaneous detection of 40 + protein markers within a single tissue section, preserving spatial information and enabling detailed analysis of tissue architecture and cellular interactions. MIBI is particularly useful in immuno-oncology and immunology, where it can reveal complex cellular environments and signaling networks within tumors or immune tissues [89].

**Imaging Mass Cytometry (IMC**) approach combines the principles of mass cytometry and imaging to enable high-dimensional, spatially resolved protein analysis within tissues. IMC uses metal-tagged antibodies, similar to MIBI, but the detection process is different. In IMC, a laser ablates the tissue, and the vaporized particles are carried into a mass cytometer, where the metal tags are quantified. This process allows for the simultaneous imaging of over 40 protein markers within a single tissue section. IMC is particularly useful for studying the tumor microenvironment, immune cell interactions, and the architecture of complex tissues. It provides a detailed view of protein expression patterns while maintaining the spatial context, making it an important tool for research in cancer, immunology, and neuroscience [90].

**CODEX (CO-Detection by indEXing)** is an advanced multiplexed imaging technology that enables the simultaneous detection of dozens of protein markers at single-cell resolution within complex tissue environments. Developed by Akoya Biosciences, CODEX employs a unique barcoding system combined with iterative rounds of fluorescent imaging to achieve high-dimensional spatial proteomics. The technology works by using DNA-barcoded antibodies that bind to specific protein targets in the tissue. After binding, fluorescently labelled complementary oligonucleotides are introduced, allowing the visualization of the protein of interest. The tissue is then imaged, the fluorescent signal is removed, and the process is repeated for multiple rounds, each time detecting a different set of protein markers. This iterative process can resolve spatial patterns of protein expression across entire tissue sections, providing insights into cellular interactions, tissue architecture, and the microenvironment’s role in disease [91, 92].

These technologies represent significant advancements in spatial proteomics, offering enhanced resolution and the ability to analyze complex proteomic landscapes at cellular and subcellular levels. They provide critical insights that are essential for advancing our understanding of diseases like cancer, where the spatial organization of proteins plays a pivotal role in disease progression and therapeutic response.

**Table 1:** Comprehensive examination of various spatial transcriptomic techniques, exploring into their mechanisms, operational principles, and methodological approaches, while discerning and addressing the strengths and weaknesses inherent in each method

Spatial transcriptomic investigation methods	
**1. NanoString GeoMx Digital Spatial Profiler (DSP):**	
**How it Works**: Utilizes a spatial barcoding technique where unique barcodes are attached to individual mRNA or protein molecules, allowing their identification during downstream analysis. The tissue section is imaged, and the barcoded molecules are counted, providing a spatially resolved readout of gene expression or protein distribution.	
**Strengths**:	**Weaknesses**:
**High Multiplexing Capability**: It allows for the simultaneous analysis of hundreds of protein or RNA targets within a single tissue section, enabling comprehensive profiling of complex tissues. **Spatial Resolution**: The platform offers high spatial resolution, allowing researchers to precisely localize gene or protein expression within specific regions of interest, such as tumor margins or specific cell populations. **Flexibility**: It can analyze both FFPE and fresh frozen tissue and BM trephine samples, making it versatile and compatible with a wide range of biological samples. **Quantitative and Reproducible Data**: The technology provides quantitative, reproducible data that can be easily integrated with other omics data, offering robust insights into tissue biology. **Customizable Regions of Interest**: Researchers can select specific regions within a tissue for detailed analysis, enabling targeted studies on particular areas of interest, such as tumor microenvironments or regions of fibrosis.	**Limited Subcellular Resolution**: While GeoMx DSP offers high spatial resolution, it does not achieve the subcellular resolution provided by some other technologies, which may limit its ability to resolve highly localized molecular events within cells. **Data Complexity and Analysis**: The large and complex datasets generated require advanced bioinformatics tools and expertise for proper analysis, which can be a barrier for some researchers. **High Cost**: The technology and its associated reagents are expensive, which may limit accessibility for smaller labs or those with limited budgets. **Long Processing Times**: The process of capturing and analyzing data can be time-consuming, especially when dealing with large numbers of targets or samples, potentially delaying results. **Dependency on Predefined Panels**: While customizable, the technology often relies on predefined panels of probes or antibodies, which may limit the ability to explore novel or less well-characterized targets.
**2. Hybridization-based In Situ Sequencing (HybISS)**:	
**How it works**: combines in situ hybridization with sequencing. In this method, tissue sections are subjected to rounds of hybridization with sets of fluorophore-labeled probes. Following each round, the fluorophores are imaged, and the associated RNA sequences are sequenced directly in situ. This approach allows for the visualization and sequencing of RNA transcripts within intact tissues.	
**Strengths**:	**Weaknesses**:
**Direct visualization**: RNA detected in intact tissues preserves spatial context. **Spatially resolved**: sequencing enhances understanding of gene expression patterns.	**Limited throughput**: restricted analysis to a smaller number of genes. **Multiplexing**: challenges in achieving high multiplexing may limit simultaneous analysis.
**3. SEQ-FISH (Sequential Fluorescent In Situ Hybridization)**:	
**How it Works**: it employs combinatorial fluorescence in situ hybridization. Multiple rounds of hybridization are performed with unique sets of fluorophore-labeled probes, each targeting specific RNA sequences. The resulting fluorescence signals from each round are sequentially imaged, allowing for the visualization of individual RNA molecules with high multiplexing.	
**Strengths**:	**Weaknesses**:
**Multiplexing**: High multiplexing capabilities, providing the ability to visualize numerous RNA targets. **Subcellular resolution**: allowing for detailed insights into cellular and molecular organization. **Sensitivity**: Visualization of individual RNA molecules enhances sensitivity.	**Not user-friendly**: Labor-intensive methodology, requiring careful optimization and extensive manual handling. **Sensitivity**: challenges in quantification and potential signal overlap may impact data interpretation.
**4. CosMX Spatial Molecular Imager (SMI)**	
**How it Works**: it uses gene-specific probes to hybridize with RNA in tissue sections, followed by iterative rounds of fluorescent imaging to detect and quantify RNA and protein targets at subcellular resolution. This method preserves the spatial context of gene expression, providing detailed molecular insights within the native tissue environment.	
**Strengths**:	**Weaknesses**:
**High-Resolution Spatial Mapping**: It allows for detailed subcellular resolution of gene expression, enabling researchers to visualize and analyze the spatial distribution of thousands of RNA and protein targets within their native tissue context. **Multiplexing Capability**: The platform’s ability to simultaneously detect and quantify a large number of targets in a single sample provides a comprehensive view of the molecular landscape, which is essential for understanding complex tissue environments. **Preservation of Tissue Architecture**: By maintaining the spatial integrity of the tissue, CosMX enables the study of cellular interactions and microenvironmental influences, which are critical for understanding disease mechanisms in situ. **Integration with Bioinformatics**: The spatial data generated can be integrated with downstream bioinformatics analyses to identify pathways, networks, and cellular interactions, providing deeper insights into the molecular underpinnings of diseases. **Versatility**: CosMX is compatible with both FFPE and fresh frozen tissue samples, making it versatile for various types of research, including studies on archived clinical samples.	**Complexity of Data Analysis**: The high level of detail and large datasets generated require sophisticated bioinformatics tools and expertise for analysis, which can be a barrier for some researchers. **High Cost**: The technology and associated reagents are expensive, which may limit accessibility for some labs, particularly those with limited funding. **Technical Expertise Required**: Operating the CosMX platform and interpreting its data require specialized training and experience, making it less accessible to labs without such expertise. **Potential for Signal Overlap**: Despite advanced imaging techniques, the use of multiple fluorophores in high-multiplex settings can lead to signal overlap, potentially complicating data interpretation. **Sample Preparation Sensitivity**: The quality of data is highly dependent on the quality of the tissue sample and the precision of the sample preparation process, making it critical to optimize these steps to avoid variability in results.
**5. Slide-seq/Slide-seqV2**:	
**How it Works**: It utilizes spatial barcoding to link transcripts to specific spatial locations on a tissue section. mRNA is captured on a surface of microscopic beads, and the spatial origin is determined by the attached barcode.	
**Strengths**:	**Weaknesses**:
**High Throughput**: It allows for the simultaneous analysis of thousands of spatially resolved transcriptomic profiles. **Versatility**: Can be applied to various tissues and samples.	**Resolution Limitations**: While high throughput, the spatial resolution might not match that of other methods. **Sensitivity to Sample Size**: Performance may vary depending on the size and type of the sample.
**6. Xenium by 10x Genomics**	
**How it works**: It works by hybridizing tissue sections with gene-specific probes that bind to target RNA molecules. These probes are then visualized through fluorescence imaging, capturing the spatial distribution of transcripts at single-cell resolution. The RNA is subsequently sequenced, providing a comprehensive gene expression profile within the tissue’s spatial context.	
**Strengths**:	**Weaknesses**:
**High Spatial Resolution**: Xenium provides single-cell resolution, allowing for detailed mapping of gene expression across complex tissue environments. This high spatial resolution is crucial for understanding cellular interactions within tissues. **Comprehensive Transcriptome Coverage**: The platform captures thousands of RNA molecules in situ, providing a broad and detailed gene expression profile. This makes it suitable for complex studies requiring comprehensive transcriptomic data. **Preservation of Spatial Context**: It maintains the spatial organization of tissues, enabling researchers to study the precise location of gene expression relative to tissue architecture. This is particularly valuable in understanding how cellular positioning affects function and disease progression. **Scalability and High Throughput**: Xenium is designed to handle large tissue sections and a high number of samples, making it suitable for large-scale studies. Its high throughput capabilities allow for the processing of multiple samples simultaneously. **Versatility Across Applications**: It is applicable in various research fields, including oncology, neuroscience, and immunology, making it a versatile tool for investigating different biological systems and diseases.	**Complex Data Analysis**: The high volume and complexity of data generated require advanced bioinformatics tools and expertise. This can be a barrier for some researchers, especially those without access to specialized computational resources. **Cost**: As with many advanced sequencing and imaging platforms, the cost of Xenium, including reagents and data processing, can be high. This may limit accessibility for smaller labs or those with limited funding. **Sample Preparation Sensitivity**: The quality of the data is highly dependent on the quality of the tissue samples and the precision of the sample preparation process. Suboptimal sample handling can lead to artifacts or incomplete data. **Long Processing Time**: The comprehensive nature of the analysis, which includes both imaging and sequencing, can result in longer processing times compared to simpler gene expression profiling methods. **Integration with Other Data Types**: While Xenium provides robust spatial transcriptomic data, integrating this data with other types of omics data (e.g., proteomics, metabolomics) requires additional tools and expertise, which can complicate the overall workflow
**7. 10x Genomics Visium Spatial Gene Expression**:	
**How it Works**: It integrates spatial barcoding with RNA sequencing to capture transcriptomic information while maintaining spatial context. Tissue sections are placed on a capture slide, and transcripts are barcoded before sequencing.	
**Strengths**:	**Weaknesses**:
**High Spatial Resolution**: It provides detailed spatial resolution, enabling the localization of gene expression across entire tissue sections, which is crucial for understanding tissue architecture and cellular interactions. **Broad Transcriptome Coverage**: The platform captures genome-wide gene expression data, allowing researchers to profile thousands of genes simultaneously and providing a comprehensive view of the transcriptome within its spatial context. **Compatibility with Histological Staining**: Visium integrates well with traditional histological techniques, such as H&E staining, allowing for the correlation of spatial transcriptomics data with tissue morphology and pathology. **Versatility Across Sample Types**: It is compatible with both fresh frozen and FFPE tissue samples, making it versatile for various types of research, including studies on archived clinical samples. **Scalability**: The platform is scalable, allowing for the analysis of multiple tissue sections in parallel, which is advantageous for large-scale studies and comparative analyses.	**Lower Cellular Resolution**: While Visium provides spatial context, its resolution is typically at the level of clusters of cells rather than single cells, which can limit the ability to discern fine details within highly heterogeneous tissues. **Complex Data Analysis**: The large amount of data generated requires advanced bioinformatics tools and expertise for processing and interpretation, which may be a challenge for some research teams. **High Cost**: The cost of the platform, including consumables and data analysis, can be high, potentially limiting its accessibility for smaller labs or those with limited funding. **Tissue Sectioning Requirements**: The requirement for precise tissue sectioning and placement on capture slides can be technically demanding, and any errors in sample preparation may affect data quality. **Limited Single-Cell Resolution**: Although Visium captures genome-wide expression, it does not achieve the single-cell resolution provided by some other technologies, which might limit the ability to resolve complex cell types and states within small regions of tissue.

These SRT technologies offer distinct advantages in unraveling the spatial intricacies of MF. The choice among them depends on specific research objectives, spatial resolution requirements, and the nature of the information sought. The integrated insights from these technologies herald a new era in our comprehension of MF, fostering optimism for innovative therapeutic breakthroughs on the horizon.

In selecting a spatial transcriptomic technique, researchers should weigh factors such as spatial resolution, multiplexing capabilities, throughput, and the complexity of data analysis, ensuring alignment with the specific demands of their research questions and biological systems. Each technique contributes uniquely to the spatial transcriptomics landscape, offering a spectrum of tools for diverse scientific investigations (Table 2).

**Table 2:** Comparative resumé of the spatial transcriptomics investigation methods explored in the manuscript

Comparative Analysis					
Technology	Resolution	Targeted Molecules	Sample Compatibility	Strengths	Weaknesses
GeoMX (NanoString)	Multi-cell regions	1000 + of RNA or Proteins	Fresh frozen, FFPE	High multiplexing, customizable regions, quantitative data, compatible with histology	Limited subcellular resolution, complex data analysis, high cost
CosMX (NanoString)	Subcellular	1000 + RNA, 60 + Proteins	Fresh frozen, FFPE	High sensitivity, subcellular resolution, large multiplexing, simultaneous RNA and protein analysis	Complex data processing, optical crowding, long imaging times, high cost
Xenium (10x Genomics)	Single-cell	1000s of RNA	Fresh frozen, FFPE	High spatial resolution, comprehensive transcriptome coverage, single-cell analysis	High cost, complex data analysis, requires advanced computational resources
Visium (10x Genomics)	Multi-cell clusters	1000s of RNA	Fresh frozen, FFPE	Broad transcriptome coverage, compatible with histology, scalable for large studies	Limited resolution (not single-cell), complex data analysis, high cost
Slide-seq / Slide-seqV2	Single-cell	1000s of RNA	Fresh frozen	High spatial resolution, cost-effective, scalable to larger areas	Requires expertise in bead preparation and alignment, lower sensitivity than other methods
Hybridization-based In Situ Sequencing (HybISS)	Subcellular	100s to 1000s of RNA	Fresh frozen, FFPE	High spatial resolution, high multiplexing, good sensitivity	Limited to predefined probes, complex hybridization steps, potential for cross-reactivity
SEQ-FISH (Sequential FISH)	Subcellular	100s to 1000s of RNA	Fresh frozen	Extremely high multiplexing, single-molecule sensitivity, subcellular resolution	Requires extensive optimization, complex imaging and data analysis, high cost of fluorophores
**Resolution**: Ranges from multi-cell regions (GeoMX) to subcellular (CosMX, HybISS, SEQ-FISH) and single-cell (Xenium, Slide-seq/Slide-seqV2).					
**Sample Compatibility**: Most technologies work with both fresh frozen and FFPE samples, though some are more specialized (Slide-seq primarily uses fresh frozen).					
**Targeted Molecules**: All platforms can analyze RNA, with some (GeoMX, CosMX) also targeting proteins.					
**Strengths**: Includes high spatial resolution, broad multiplexing capabilities, and compatibility with histological techniques.					
**Weaknesses**: Common challenges include complex data analysis, high cost, and technical complexity in sample preparation and imaging.					

## Spatially resolved transcriptomic in myelofibrosis

SRT, as already mentioned as a revolutionary advancement in contemporary molecular biology, stands as an indispensable tool for unraveling the intricate spatial complexities inherent in MF within both the BM and spleen microenvironments [93]. By harnessing the synergy between spatially resolved high-throughput sequencing and microscopic techniques, SRT facilitates a distinct dissection of the cellular and molecular landscapes, providing unparalleled insights into the heterogeneity characterizing MF pathogenesis. This discourse inquiries into the assorted applications of SRT in scrutinizing MF, elucidating its utility in spatial resolution, cellular profiling, molecular signatures, identification of niche alterations, understanding disease progression, integration with clinical data, and the formulation of targeted therapeutic approaches.

The profound impact of SRT transcends conventional boundaries in spatial molecular profiling, thereby leading off for a deeper understanding of MF at unprecedented levels of resolution and specificity. SRT’s utility is multifold, starting with its capacity to achieve high-resolution spatial mapping within the BM, revealing heterogeneity across various cell types, including hematopoietic cells, fibroblasts, and immune cells. Similarly, SRT extends its capabilities to the spleen, characterizing spatial heterogeneity and exploring the distribution of abnormal hematopoietic cells and fibrotic changes.

In terms of cellular profiling, SRT identifies and characterizes diverse cell populations within the BM, offering insights into the spatial distribution of hematopoietic stem cells, progenitor cells, fibroblasts, and immune cells [94, 95]. In the spleen, SRT contributes to elucidating the extent of abnormal cell infiltration, including the distribution of hematopoietic cells contributing to splenomegaly. SRT’s prowess in unveiling molecular signatures is exemplified by its ability to simultaneously profile multiple genes, exposing molecular signatures associated with fibrotic areas, altered hematopoiesis, and deregulated signaling pathways within the BM. The spleen, under SRT scrutiny, reveals spatially regulated genes linked to fibrosis, potentially uncovering molecular drivers of spleen enlargement and abnormal cell accumulation.

Additionally, SRT can provide insights into spatial alterations in the BM niche, including changes in cytokine signaling, ECM composition, and interactions with stromal cells, which can recur in the spleen, and SRT can aid in identifying microenvironmental changes contributing to observed pathological features.

The ability of SRT to map the spatial progression of MF within the BM allows for the identification of regions with different disease stages and the understanding the spatial characteristics of the spleen, a major site of blood cell sequestration, may offer insights into disease severity and progression [39].

Integration of SRT data with clinical parameters enables the correlation of spatial molecular profiles with patient outcomes, disease severity, and treatment responses. SRT may unveil spatially regulated biomarkers, providing prognostic insights and guiding treatment decisions.

Finally, SRT can reveal spatially regulated genes that may serve as potential therapeutic targets for MF, facilitating the development of targeted interventions. Understanding spatial heterogeneity contributes to the formulation of personalized treatment strategies, acknowledging variations in the disease microenvironment. In conclusion, SRT’s prowess in spatially resolved molecular profiling provides a comprehensive understanding of MF pathophysiology, opening avenues for targeted therapies and personalized interventions guided by the spatial intricacies of the disease microenvironment (Table 3).

**Table 3:** This table highlights the key roles of SRT in studying MF, comparing its applications in both the BM and spleen. It covers aspects such as spatial resolution, cellular profiling, molecular signatures, niche changes, disease progression, integration with clinical data, and the identification of targeted therapeutic approaches. The comparison provides insights into how SRT can be used to understand disease pathology, progression, and guide personalized treatment strategies for MF in these critical tissues

Comparative Applications of Spatially Resolved Transcriptomics in Myelofibrosis for Bone Marrow and Spleen		
Aspect	Bone Marrow	Spleen
Spatial Resolution	SRT achieves high-resolution spatial mapping in the BM, elucidating heterogeneity across hematopoietic, fibroblast, and immune cell populations.	SRT characterizes spatial heterogeneity in the spleen, detailing the distribution of abnormal hematopoietic cells and fibrotic changes.
Cellular Profiling	SRT identifies diverse BM cell populations, offering insights into the spatial distribution of stem cells, progenitors, fibroblasts, and immune cells.	SRT helps elucidate abnormal cell infiltration in the spleen, mapping the distribution of hematopoietic cells contributing to splenomegaly.
Molecular Signatures	SRT enables the simultaneous profiling of multiple genes in BM, revealing molecular signatures tied to fibrosis, altered hematopoiesis, and signaling dysregulation.	In the spleen, SRT uncovers spatially regulated genes related to fibrosis and abnormal cell accumulation, identifying potential molecular drivers of splenomegaly.
Identification of Niche Changes	SRT provides insights into spatial alterations in the BM niche, including changes in cytokine signaling, ECM composition, and stromal cell interactions.	SRT identifies changes in the splenic microenvironment, highlighting the factors contributing to pathological fibrosis and abnormal hematopoiesis.
Disease Progression and Subtypes	SRT maps spatial progression in the BM, identifying regions associated with distinct stages of MF.	SRT characterizes the spatial organization of disease severity and progression in the spleen, a major site of blood cell sequestration.
Integration with Clinical Data	Integrating SRT data with clinical outcomes allows researchers to correlate spatial molecular profiles with disease severity, prognosis, and treatment responses.	SRT could reveal spatially regulated biomarkers in the spleen, offering prognostic value and guiding personalized treatment approaches.
Targeted Therapeutic Approaches	SRT identifies spatially regulated genes in the BM that could serve as therapeutic targets, facilitating the development of targeted treatments.	SRT’s understanding of spatial heterogeneity in the spleen helps formulate personalized treatment strategies, addressing the microenvironment’s variations.

## Conclusions

SRT is revolutionizing the study MF by providing a detailed view of the spatial organization of gene expression within tissues. This technology holds immense potential not only for understanding the disease’s pathophysiology but also for improving diagnosis, prognosis, and tailoring treatment strategies.

In the context of MF, traditional **diagnostic approaches** often rely on histopathological evaluation of BM biopsies, supplemented by molecular testing for known driver mutations such as *JAK2*,* CALR*, and *MPL*. While these methods are effective, they lack the ability to capture the spatial heterogeneity of gene expression within the BM microenvironment. SRT can bridge this gap by providing a comprehensive spatial map of gene expression at the single-cell level within BM biopsies. SRT could be integrated into the diagnostic workflow to identify specific spatial gene expression patterns that correlate with early fibrotic changes, even before they are detectable by conventional histology. For instance, the detection of localized upregulation of pro-fibrotic genes in specific niches within the BM could serve as an early marker of disease, allowing for earlier intervention.

Moreover, SRT could help distinguish between different subtypes of MPNs by identifying unique spatial transcriptomic signatures. This could be particularly useful in cases where the morphological distinction between MF and other MPNs, such as Essential Thrombocythemia or Polycythemia Vera, is challenging.

SRT also offers significant **prognostic** potential by enabling the identification of spatially distinct gene expression patterns that correlate with disease progression and outcome. For example, the spatial distribution of malignant clones and their interaction with the BM stroma could provide insights into the aggressiveness of the disease. SRT could be used to develop a prognostic scoring system based on the spatial organization of gene expression within the BM. This system could integrate information about the spatial proximity of malignant clones to pro-fibrotic stromal cells, the extent of BM fibrosis, and the presence of specific inflammatory microenvironments that support disease progression. Patients with high-risk spatial profiles could be identified early and monitored more closely, allowing for timely intervention.

Recent studies have demonstrated that specific spatial patterns of gene expression are associated with a higher likelihood of leukemic transformation in MF. For instance, regions within the BM with a high concentration of inflammatory cytokine expression and altered extracellular matrix components have been linked to poor prognosis [96]. By integrating SRT into prognostic models, clinicians could more accurately predict disease trajectory and tailor treatment accordingly.

One of the most promising applications of SRT in MF is its potential to guide **personalized treatment strategies**. Currently, treatment options for MF include JAK inhibitors, targeted therapies, and allo-HSCT. However, patient responses to these treatments are highly variable, and there is a need for better tools to match patients with the most effective therapies. SRT could be used to map the spatial distribution of drug target expression within the BM and spleen, enabling the identification of patients who are most likely to benefit from specific treatments. For example, patients with a spatially localized overexpression of JAK2 within certain BM niches might respond better to JAK inhibitors, while those with a broader distribution of inflammatory signals might benefit from combination therapies targeting multiple pathways.

Furthermore, SRT could help in monitoring treatment response by tracking changes in the spatial organization of gene expression over time. This would allow for the early detection of treatment resistance, enabling clinicians to adjust therapy before the disease progresses. For instance, a shift in the spatial expression pattern of pro-survival genes could indicate the emergence of resistant clones, prompting a change in treatment strategy.

*Ex his omnibus* the conclusions drawn from diverse facets of MF a comprehensive understanding of this intricate disorder emerges. MF intricately involves the dysregulation of hematopoietic and stromal cells within the BM microenvironment. The complexities span abnormalities in the BM niche, contributing to the observed fibrosis, altered immune responses, and cytokine deregulation. Unraveling the intricacies of disease pathology and the microenvironment stands as a cornerstone for advancing therapeutic strategies and elevating patient outcomes. The ongoing trajectory of research continues to consider the molecular and cellular mechanisms orchestrating MF, offering a promising foundation for the development of innovative therapeutic interventions.

Turning attention to the spleen, a pivotal organ affected by the altered BM microenvironment in MF, profound changes unfold. Spleen enlargement transcends a mere consequence of EMH, instead embodying a complex interplay involving fibrosis, abnormal cell infiltration, and pathological transformations. The metaphorical designation of the spleen as a “tumor surrogate” underscores its substantial impact on the disease process, contributing significantly to the manifestation of symptoms and complications in MF. A profound understanding of these dynamic interactions becomes imperative for managing the clinical facets of MF and formulating targeted therapeutic strategies that address the intricate pathophysiology of the spleen in the disease context.

Introducing SRT into this narrative unveils a transformative potential in providing a comprehensive and spatially resolved comprehension of MF within both the BM and spleen. By meticulously dissecting molecular and cellular features in specific regions, SRT contributes to a deeper understanding of the disease landscape, enriching research endeavors and guiding potential therapeutic interventions. As a technological marvel, SRT offers a sophisticated lens into the spatial heterogeneity of MF, transcending traditional methodologies and providing a foundation for tailored and precise approaches to both research and clinical management. The integration of insights from MF research, spleen dynamics, and SRT heralds a new era in our understanding of this complex disorder, fostering optimism for innovative therapeutic breakthroughs on the horizon.

## Future perspectives

### Targeting the microenvironment

SRT could identify specific stromal cell populations within the BM and spleen that play a crucial role in supporting malignant hematopoiesis. By targeting these stromal cells with novel therapies, it may be possible to disrupt the microenvironmental support for the malignant clones, thereby halting disease progression.

### Spatial drug delivery

Leveraging SRT, future therapies could be designed to deliver drugs directly to the spatial regions within the BM or spleen where malignant cells are most active. This would maximize therapeutic efficacy while minimizing systemic side effects.

### Combination therapies

SRT could identify regions within the BM or spleen where multiple pathways are activated simultaneously, suggesting a rationale for combination therapies that target different components of the disease. For example, combining JAK inhibitors with anti-fibrotic agents in patients with a specific spatial profile could yield better outcomes than monotherapy.

SRT is poised to transform the management of MF by offering deeper insights into the spatial organization of gene expression within the BM and spleen. By enhancing our understanding of the disease’s spatial biology, SRT can improve diagnosis, refine prognostic assessments, and guide personalized treatment strategies. As this technology continues to advance, it holds the potential to not only enhance clinical outcomes for patients with MF but also to open new avenues for research and therapeutic development.

## Data Availability

No datasets were generated or analysed during the current study.
